# Perspective: Aligned arrays of electrospun nanofibers for directing cell migration

**DOI:** 10.1063/1.5058083

**Published:** 2018-12-10

**Authors:** Jiajia Xue, Tong Wu, Younan Xia

**Affiliations:** 1 The Wallace H. Coulter Department of Biomedical Engineering, Georgia Institute of Technology and Emory University, Atlanta, Georgia 30332, USA; 2 School of Chemistry and Biochemistry, Georgia Institute of Technology, Atlanta, Georgia 30332, USA; 3 School of Chemical and Biomolecular Engineering, Georgia Institute of Technology, Atlanta, Georgia 30332, USA

## Abstract

Cell migration plays an important role in a wide variety of biological processes, including embryogenesis, wound healing, inflammation, cancer metastasis, and tissue repair. Electrospun nanofibers have been extensively explored as scaffolds to manipulate cell migration owing to their unique characteristics in mimicking the hierarchical architecture of extracellular matrix. In particular, aligned arrays of electrospun nanofibers are capable of guiding and promoting the directional migration of cells. The physical parameters and properties of the aligned nanofibers, including their size, modulus, and surface chemistry, can all affect the migratory behaviors of cells, while the controlled release of growth factors and drugs from the nanofibers can also be utilized to influence cell migration. By manipulating cell migration, electrospun nanofibers have been applied to promote tissue repair and help eradicate tumors *in vivo*. In this perspective, we highlight recent developments in collecting electrospun nanofibers as aligned arrays and then illustrate how the aligned nanofibers can be utilized to manipulate cell migration.

## I. INTRODUCTION

Cell migration plays a central role in a wide variety of biological processes, as exemplified in embryogenesis, inflammatory responses, wound healing, and tissue repair.^1–4^ In the case of tissue repair, for example, the recruitment and migration of desirable cells to the defect site is key to the construction and regeneration process. For instance, the regeneration of skin includes re-establishment of the dermis structure, which can be accelerated by triggering the directional migration of epithelial cells and fibroblasts to the wound area.^2^ On the other hand, the deregulation of cell migration can lead to pathological conditions such as impaired healing and cancer metastasis.^5–7^

**FIG. 1.**
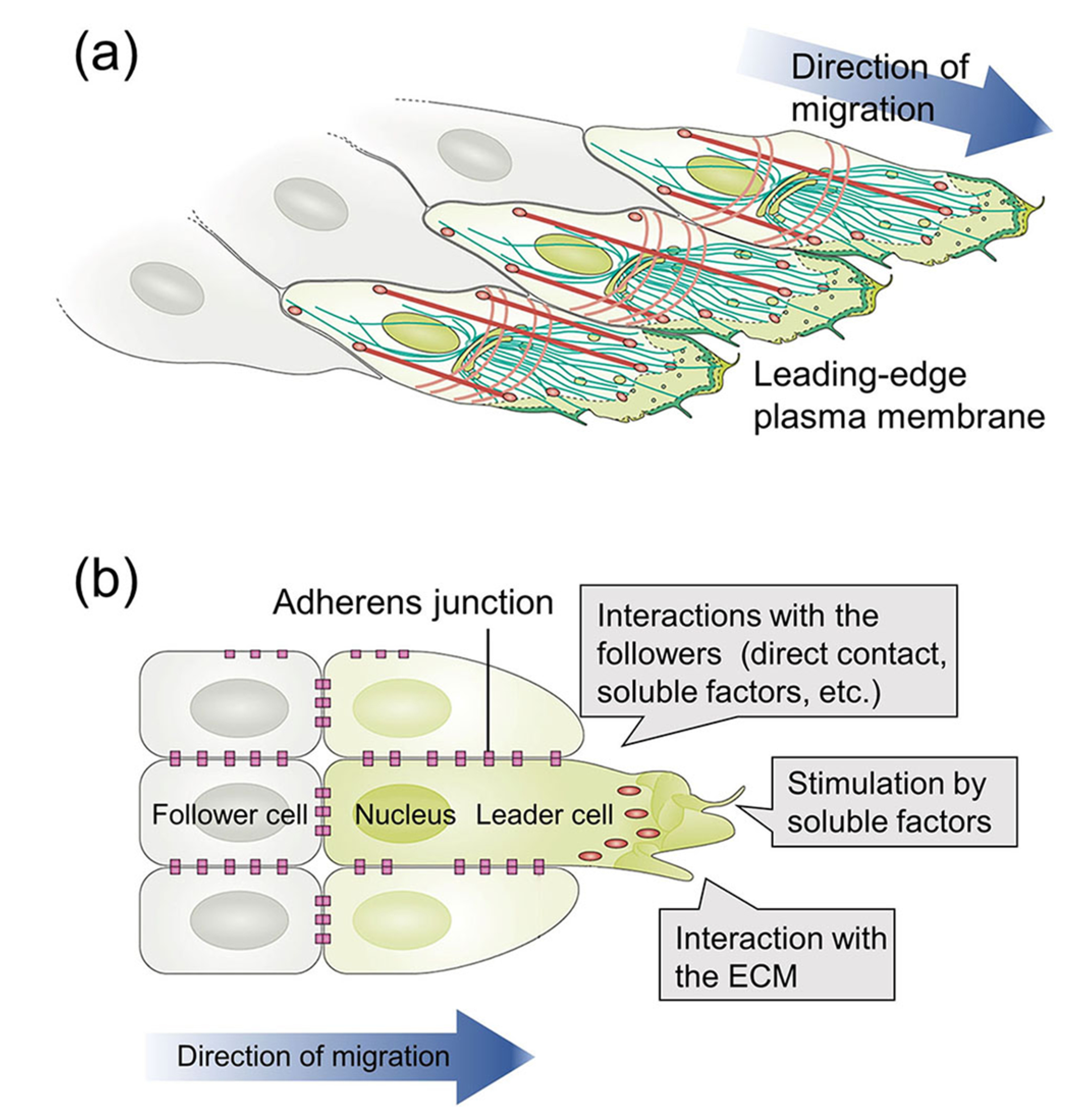
(a) The molecular mechanism of cell migration and (b) cell-cell communication during collective cell migration.^10^ Reprinted with permission from Mayor and Etienne-Manneville, Nat. Rev. Mol. Cell Biol. **17**, 97 (2016). Copyright 2016 Springer Nature.

Cell migration is a dedicated process that involves cell adhesion, polarization, and forward movement, and its direction and speed are regulated by the intricate interactions between the cells and their microenvironments.^8–10^ Figure 1 shows the molecular mechanism that controls the collective migration of cells.^10^ The leader cells are polarized under stimulation by interacting with the underlying substrate and surrounding soluble factors such as growth factors and drugs. At the front of each cell, cytoskeletal rearrangements lead to the formation of membrane protrusions such as filopodia and lamellipodia, serving as the main engine for movement. The microtubule network and components of the intracellular membrane also organize in a polarized manner along the direction of migration, as illustrated in Fig. 1(a). The leading edge of the cell is then stabilized via adhesion to the substrate. Cell-cell communication, both between followers and between follower and leader cells, further improves the collective movement of the cells [Fig. 1(b)]. In response to asymmetric environmental factors, the cells undergo directed migration. Different types of directed cell migration have been observed, including haptotaxis, chemotaxis, durotaxis, and electrotaxis.^7,11^ In principle, the types and locations of cues determine which type of directed cell migration is engaged.^12^ For instance, haptotaxis refers to cell migration toward a substrate-bound agent, whereas chemotaxis involves directed migration toward a soluble cue in the environment. Durotaxis describes cell migration in response to mechanical signals, and electrotaxis is the response to an electric field. With a growing understanding of the mechanism involved in cell migration, there is an increased effort to develop substrates capable of recapitulating key features of the ECM, which directly controls the migration of cells.

Various types of micro/nano-structured surfaces or substrates have been fabricated to manipulate the migration of cells. The most prominent technologies include soft lithography, nanolithography (e.g., writing with an e-beam or dip pen), and electrospinning.^13^ Specifically, microfluidic devices with various types of channels have been fabricated using soft lithography. The microfluidic channels are able to physically confine cells and provide guidance to their migration.^14^ The channels can also be coated or filled with ECM proteins to generate bioactive cues or integrated with protein gradients to offer chemoattractant for controlling the migration of cells.^15^ With the use of multiple channels, parallel measurements of cell migration under different experimental conditions can be performed simultaneously.^16^ The microfluidic channels, however, cannot closely mimic the structure and composition of native ECM because they are limited in terms of materials, configurations, and feature size. On the other hand, e-beam lithography scans a focused beam of electrons across a surface covered with an electron-sensitive film or resist to generate patterned features, and it has been applied to fabricate nanostructures as small as 5 nm for controlling cell migration.^13,17^ However, e-beam writing needs a dedicated, expensive instrument, and only a limited number of materials are suitable for use as the substrates.^13^ Typically, the structures can only be patterned over a relatively small area. In comparison, electrospinning is a simple technique that offers more flexibility in terms of materials. It can be used to generate continuous fibers with diameters ranging from tens of nanometers to several micrometers and, more important, fibrous structures resembling native ECM.^18,19^ Recent progress in fabricating electrospun fibers with complex compositions (e.g., biomolecules and nanoparticles) and well-controlled alignment have made the fibers a class of attractive substrates for controlling the migration of cells. As another major advantage over the microfluidic channels and lithographically patterned surfaces, electrospun fibers can be made free-standing and further implanted as scaffolds *in vivo* to recruit cells for tissue regeneration or cancer treatment.

Electrospinning relies on the electrostatic repulsion between surface charges to draw nanofibers from a viscoelastic fluid. When a polymer solution is pumped out through a spinneret connected to a high voltage, a conical structure known as “Taylor cone” is formed, and a jet emanates from the apex of the cone toward a grounded collector. The jet continues to decrease in diameter as a result of stretching caused by electrostatic repulsion, whipping, and solvent evaporation until the jet is solidified and deposited on the collector.^18^ The properties of the as-spun nanofibers (e.g., the alignment, size, composition, and modulus) can be easily tuned to match those of native ECM by adjusting the properties of the polymer solution and the parameters of the electrospinning process. The surfaces of the nanofibers can also be functionalized with ECM proteins or bioactive agents through post-treatment. By mimicking native ECM in this way, electrospun nanofibers are able to manipulate the migration of cells ranging from normal cells to stem cells and tumor cells. Additionally, when the nanofibers are assembled as aligned arrays, they can guide and promote the directional migration of cells. Furthermore, due to contact guidance, the cytoskeletons of cells on the aligned nanofibers also show aligned architectures, causing the cells to produce a highly aligned collagen matrix, which is desirable for repairing tissues with anisotropic anatomies such as tendon, cardiac, and nerve tissues.^20,21^ The understanding of how nanofibers affect cell migration has progressed substantially in recent years. In this perspective, we highlight both recent findings and future expectations in regulating cell migration by aligned arrays of electrospun nanofibers.

## II. ALIGNMENT OF ELECTROSPUN NANOFIBERS

**FIG. 2.**
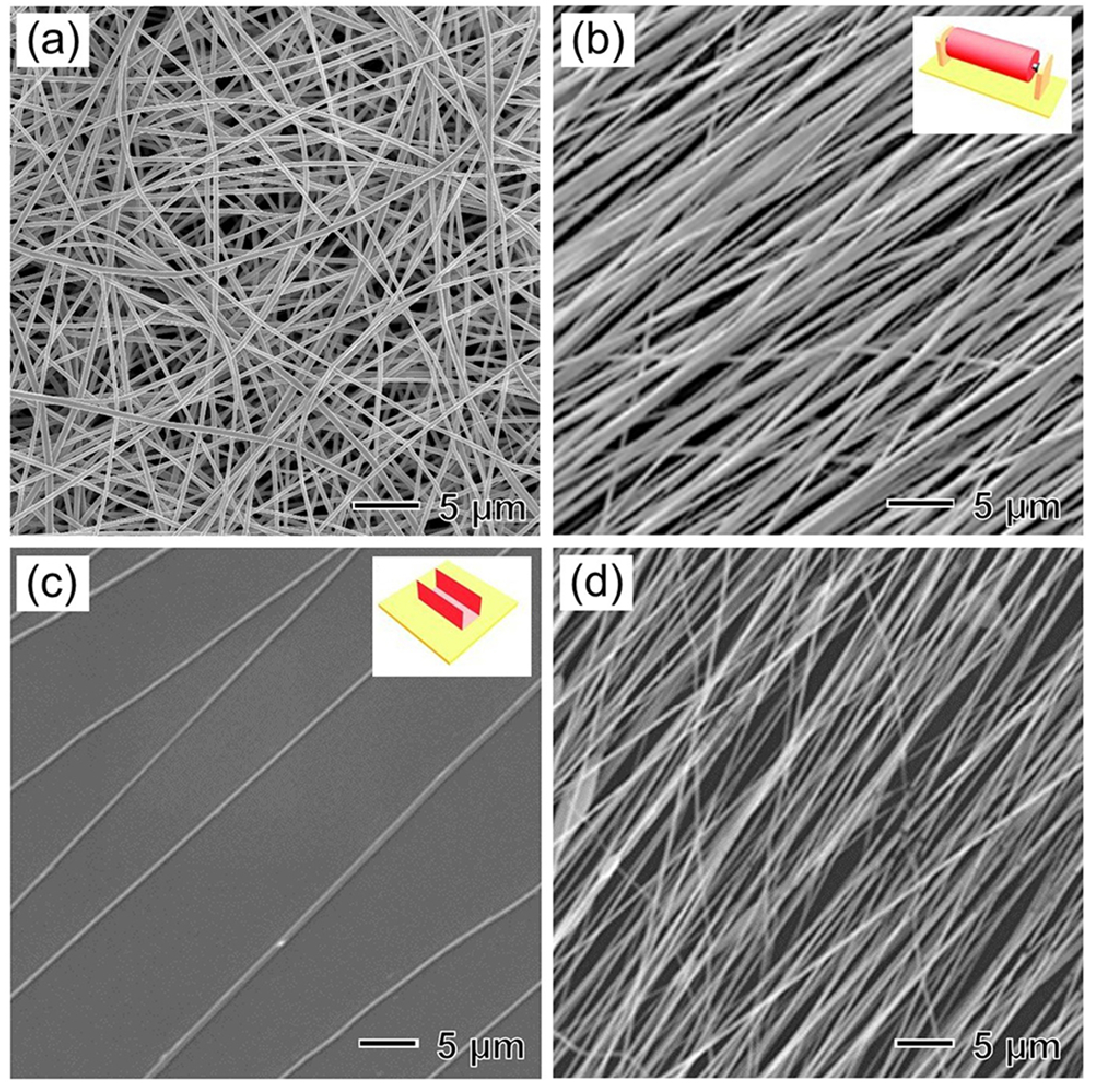
SEM images of (a) random PCL nanofibers collected on a flat conductive collector, (b) uniaxially aligned PLA nanofibers collected on a high-speed rotating mandrel (as illustrated in the inset in the top-right corner),^23^ and uniaxially aligned PCL nanofibers collected for (c) 1 min and (d) 15 min on top of the gap formed between a U-shaped stainless-steel frame (as illustrated in the inset in the top-right corner).^25^ (b) Reprinted with permission from Liu *et al.*, Adv. Mater. **27**, 2583 (2015). Copyright 2015 WILEY-VCH; [(c) and (d)] Xie *et al.*, ACS Nano **8**, 1878 (2014). Copyright 2014 American Chemical Society.

Due to the chaotic trajectory of an electrospinning jet, the nanofibers collected on a flat, grounded collector are typically deposited as a non-woven mat with no orientation, as shown by the scanning electron microscopy (SEM) image of polycaprolactone (PCL) nanofibers in Fig. 2(a). A number of methods have been developed to control the alignment of nanofibers; these methods can be divided into three major categories depending on the types of forces involved, that is, mechanical, electrostatic, and magnetic forces.^20,22^

Uniaxially aligned nanofibers can be produced using a high-speed rotating mandrel [the inset in Fig. 2(b)] as the collector. Under mechanical stretching, the nanofibers orient along the rotating direction of the mandrel. Figure 2(b) shows the SEM image of uniaxially aligned poly(lactic acid) (PLA) nanofibers collected in this way.^23^ The rotating speed of the mandrel determines the degree of alignment. To obtain uniformly aligned nanofibers, the mandrel must be rotated at a speed in accordance with the velocity of the electrospinning jet.

The alignment of electrospun nanofibers can also be manipulated using an external electric field since electrostatic charges are distributed along the electrospinning jet.^24^ When two pieces of electrically conductive substrates separated by a void gap are used as the collector [the inset in Fig. 2(c)], the charged nanofibers are stretched to span across the gap and thus are deposited as a uniaxially aligned array. This technique allows for the collection of suspended nanofibers across an air gap. The aligned nanofibers can also be conveniently transferred onto other solid substrates for further applications. By controlling the collection time, the density of the nanofibers can be varied. Figures 2(c) and 2(d) show representative SEM images of uniaxially aligned PCL nanofibers collected for 1 and 15 min, respectively, on top of the gap formed between a U-shaped stainless-steel frame.^25^ This gap technique has been further developed to align nanofibers into different patterns by varying the design of the conductive substrates.

An external magnetic field can also be applied to control nanofiber alignment.^26^ Uniaxially aligned poly(*D*,*L*-lactic-co-glycolic acid) (PLGA) nanofibers were fabricated in the presence of a magnetic field generated by two parallel permanent magnets. The charged nanofibers were spun onto the collector and stretched across the gap of two opposite magnetic poles along the direction normal to the surfaces of the magnets.

Electrospinning has been successfully applied to generate aligned nanofibers from a large number of different types of natural and synthetic polymers. For *in vitro* study of cell migration, the polymers are required to have excellent biocompatibility and suitable mechanical strength to replicate the features of the native ECM. In this regard, the most commonly used materials include both synthetic [e.g., PCL, PLGA, PLA, poly(ethylene oxide), and polyurethane] and natural (e.g., gelatin, collagen, and silk fibroin) polymers. For the *in vivo* recruitment of cells, besides biocompatibility, the scaffolds made of electrospun fibers also need to offer additional properties, such as suitable biodegradation rate and mechanical property for supporting the cells. In this regard, it is challenging to find a single polymer that meets all these requirements. As a potential solution, multiple polymers are often used to generate blended or core-sheath electrospun fibers.^20^

## III. CONTROL OF CELL MIGRATION BY ALIGNED FIBERS

**FIG. 3.**
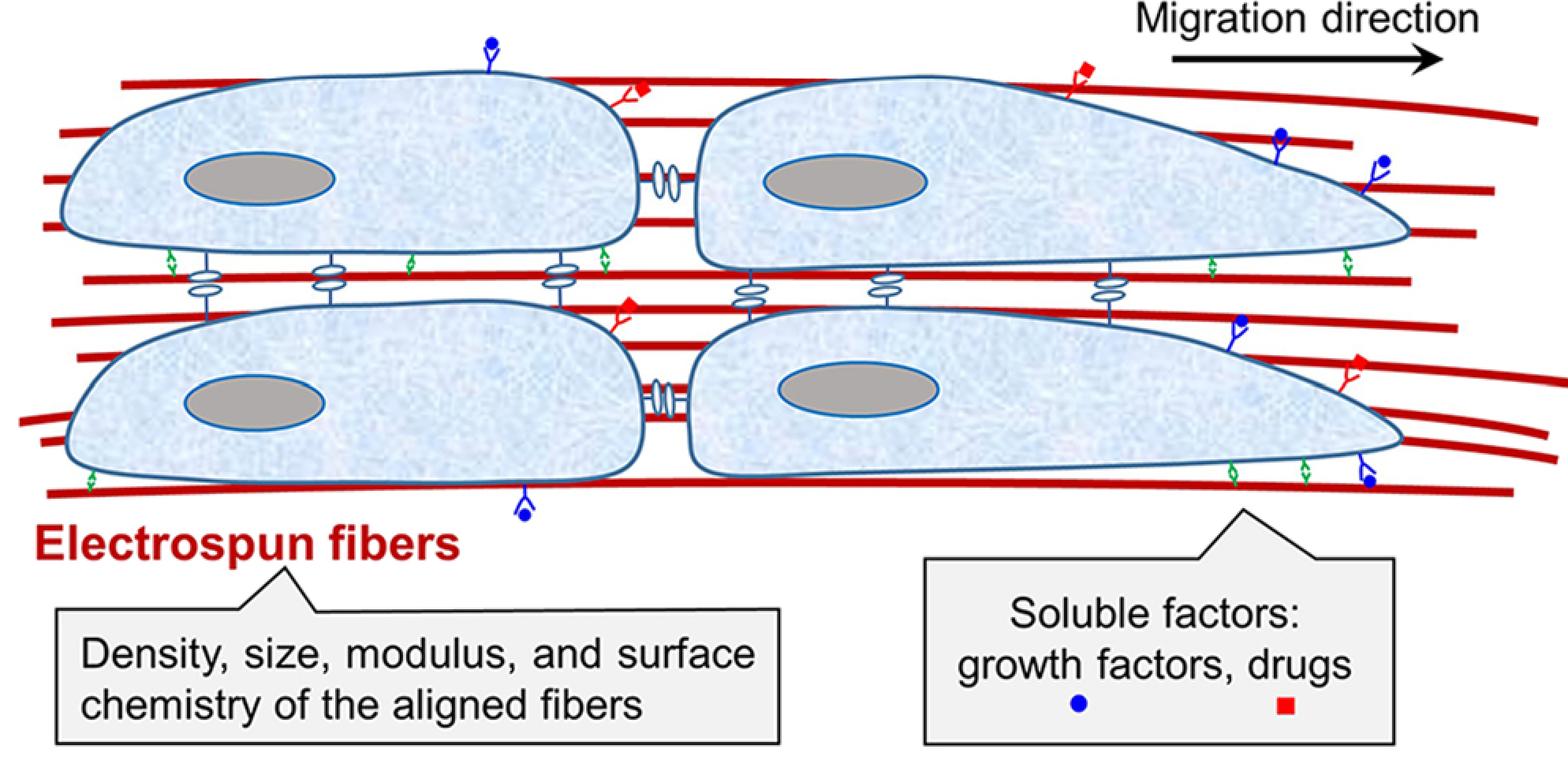
Schematic indicating that the density, size, modulus, and surface chemistry of the electrospun fibers, as well as the release of soluble factors from the fibers, can all affect the migration of cells.

As shown by the schematic in Fig. 3, the density, size, modulus, and surface chemistry of aligned fibers as well as soluble factors released from the fibers have all been explored to control the migration of cells.^27,28^ The migratory behavior of cells can be characterized by examining the distance and speed of migration over a certain period of time using metrics such as the total migration distance (total trajectory length of the cell body), linear migration speed (total migration distance divided by the total time), net migration distance (the distance from the initial to final position), effective migration speed (net migration distance divided by the total time), and migration persistence (the net migration distance divided by total migration distance of the cell, indicating the capacity of the cells to maintain the direction of motion).

### A. Migration of cells on suspended individual fibers

**FIG. 4.**
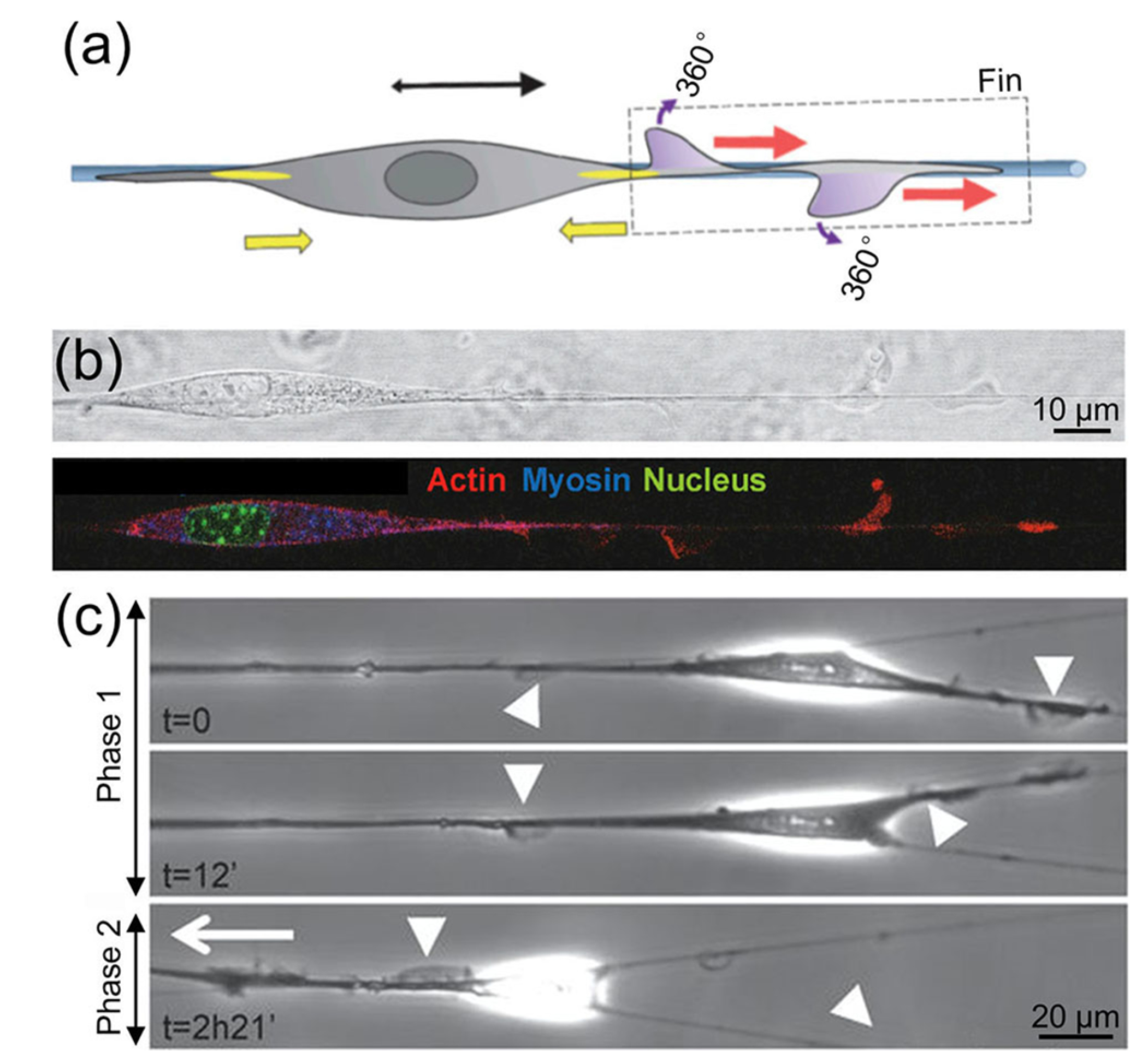
The migratory behavior of one cell on [(a) and (b)] a suspended nanofiber and (c) multiple suspended nanofibers.^29^ (a) Schematic representation and (b) micrographs showing the morphology of a 3T3 fibroblast migrating on a fibronectin-coated, suspended PCL nanofiber. The generated fin protrusions enabled the free rotational movement of the cell along the suspended nanofiber. The yellow arrows show the contribution of contractility. (c) Migration of 3T3 fibroblasts on multiple nanofibers in two phases: phase 1, no net migration with fins on both nanofibers; and phase 2, fins only on one nanofiber, leading to the migration of the cell along this nanofiber. The arrowheads indicate fin-like protrusions, and the white arrow shows the direction of migration. Reprinted with permission from Guetta-Terrier *et al.*, J. Cell Biol. **211**, 683 (2015). Copyright 2015 Rockefeller University Press.

The migratory behavior of cells on suspended individual nanofibers is distinct from that on fibrous mats consisting of densely packed nanofibers. Figure 4(a) shows a schematic representation of the morphology of a cell migrating along a fibronectin-coated, suspended PCL nanofiber.^29^ Micrographs of a 3T3 fibroblast on the nanofiber are shown in Fig. 4(b). The cell generated fin-like protrusions that were perpendicular to the main axis of cell motion and propagated toward the leading edge of the cell. The authors found that the formation of the fin was caused by the highly localized distribution of focal adhesions and actomyosin contractility. The fin protrusions allowed cell membrane extension and initiated the free rotational movement of the cell. The optimal balance between contractility [the contribution of contractility is shown by the yellow arrows in Fig. 4(a)], adhesion, and fins promoted the migration of the cell along the suspended nanofiber. On a fiber of 1.3 *μ*m in diameter, the 3T3 fibroblast displayed high directionality and migrated at a linear speed of approximately 40 *μ*m/h. When the cell encountered multiple fibers, as shown in Fig. 4(c), the fin-like protrusions dynamically probed the environment and controlled the cell’s capacity to change direction. This probing process, which was reflected by the protrusion-retraction cycle at the leading edge of the cell, occurred over a short time scale. These waves of fin-like protrusions were exhibited and utilized by a variety of cell types, including endothelia, glioma, and other fibroblast cell lines, when the cells were migrating on suspended nanofibers.

### B. The effect of fiber alignment on cell migration

On a two-dimensional mat composed of nanofibers with a high packing density rather than suspended, individual nanofibers, the fiber alignment primarily affects the migratory direction and speed of the cells. The cells rely on membrane protrusions such as filopodia and lamellipodia to move. On random nanofibers, the cells migrate randomly in all directions without preferred directionality. Each cell has a large number of junctions, resulting in small cell translocation since the cells are constrained to follow the direction of the nanofibers and thereby may change direction in a typical trajectory. However, on aligned nanofibers, due to contact guidance, the cells orient and migrate in linear paths corresponding to the direction of fiber orientation, leading to an enhancement in migration speed. Taking the migration of 3T3 fibroblasts on thermoplastic polyurethane nanofibers as an example, for a similar nanofiber diameter, the migration speed of cells cultured on uniaxially aligned nanofibers was approximately two times that of cells cultured on random nanofibers.^30^ Similar phenomena were observed for astrocytes and L929 cells on mats composed of electrospun PLA and PCL nanofibers, respectively.^31,32^ The migration of stem cells can also be accelerated by aligned nanofibers; for a similar fiber diameter, human neural progenitor cells and mesenchymal stem cells (MSCs) both showed higher migration speeds on uniaxially aligned nanofibers compared to the case of random nanofibers.^33,34^

The effect of fiber alignment on cell migration was further demonstrated in dynamic systems. A nanofibrous scaffold was electrospun from a shape-memory polymer, in which the fiber alignment could be changed on command by thermal triggering, and used to study cell migration.^35^ When the scaffold was warmed from 30 °C to 37 °C, the unidirectional alignment of the nanofibers was dynamically increased, leading to a change in cell motility from non-polarized to polarized along the fiber alignment with increased cell migration speed.

### C. The influence of fiber diameter on cell migration

**FIG. 5.**
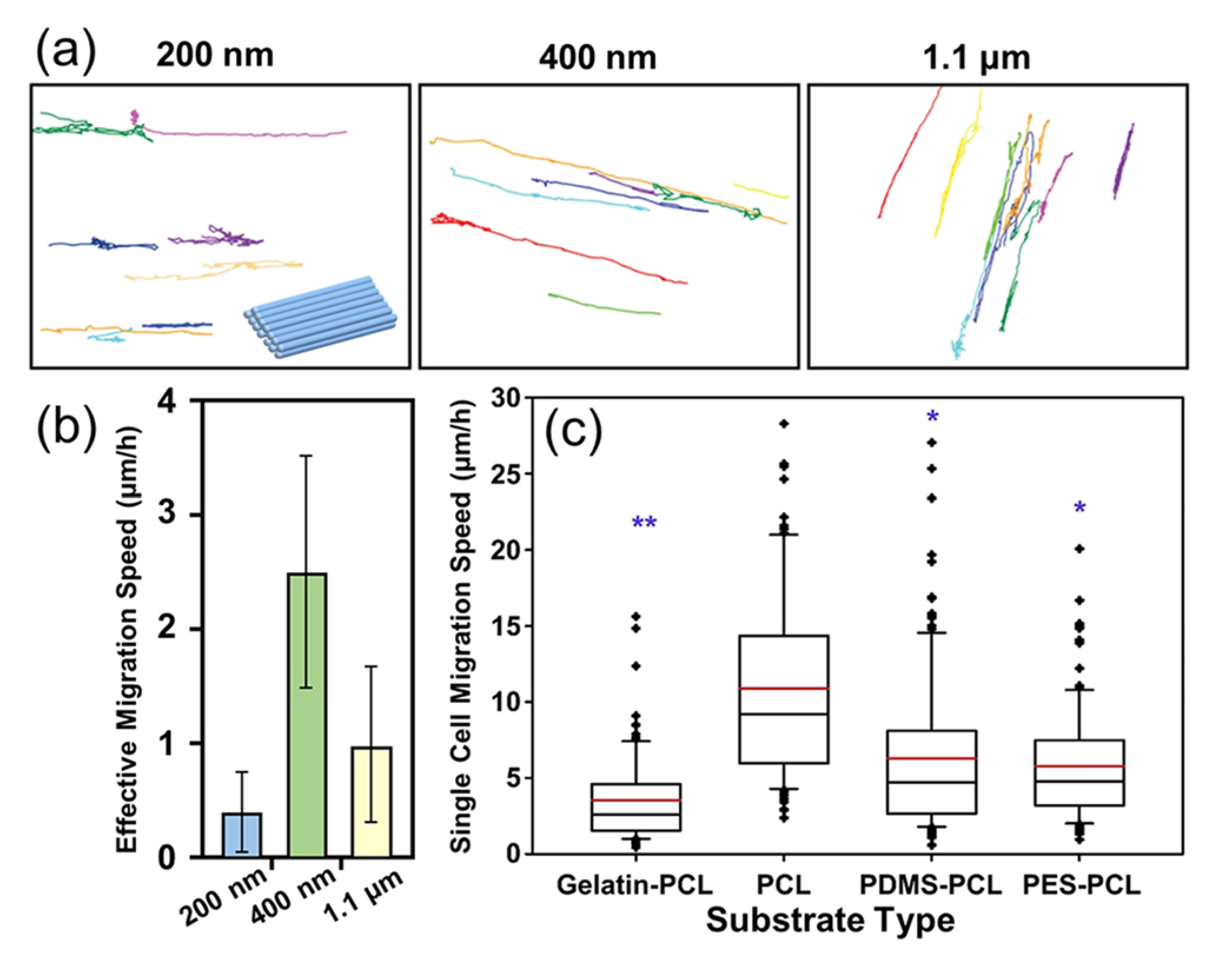
The influences of fiber diameter and modulus on the migratory behavior of cells when cultured on a fibrous mat composed of densely packed nanofibers. (a) Traces of glioblastoma cells migrating over 15–24 h on fibrous mats composed of uniaxially aligned fibers with diameters of 200 nm, 400 nm, and 1.1 *μ*m, and (b) the effective migration speeds of the cells. Effective speed was highest in cells cultured on 400-nm nanofibers.^38^ (c) The migration speed of single glioblastoma multiforme cell as a function of the fiber modulus. Nanofibers with different moduli were fabricated using the co-axial electrospinning method by changing the core material while keeping PCL as the sheath material.^45^ [(a) and (b)] Reprinted with permission from Kievit *et al.*, Adv. Healthcare Mater. **2**, 1651 (2013). Copyright 2013 WILEY-VCH; (c) Rao *et al.*, Biomaterials **34**, 5181 (2013). Copyright 2013 Elsevier.

A correlation between the fiber diameter and the migratory behavior of cells has also been reported.^36^ On uniaxially aligned PLA fibers with different diameters (large, 1325 ± 383 nm; intermediate, 759 ± 179 nm; and small, 293 ± 65 nm), the Schwann cells migrated the farthest on the large fibers and the shortest on the small nanofibers.^37^ The authors stated that the fibers with a large diameter were more densely packed than those with intermediate and small diameters. The densely packed fibers acted as barriers to impede the Schwann cells from crossing onto nearby fibers. On the intermediate and small fibers, the spacing between the fibers was large; thus, these fibers could not provide sufficient topographical cues to direct the migration of Schwann cells. However, the influence of fiber diameter on cell migration can be different depending on polymer compositions and cell types. In one study, the migratory behavior of glioblastoma cells on uniaxially aligned chitosan-PCL fibers with diameters of 200 nm, 400 nm, and 1.1 *μ*m was separately monitored.^38^ The traces of the cells migrating on the fibers with different diameters over 15–24 h are shown in Fig. 5(a). Effective cell speed was calculated by measuring the net distance that a cell traveled from its starting point and then plotting the distance over time. Based on Fig. 5(b), the nanofibers with diameters of 400 nm exhibited the highest effective cell speed of 2.5 ± 1 *μ*m/h, similar to that reported for cells invading along microvessels *in vivo*. On the aligned fibers with a diameter of 400 nm, the cells expressed higher values of invasion-related genes than those on the fibers with a diameter of 1.1 *μ*m, indicating that higher curvature of the fibers promoted the migratory behavior. In another study, uniaxially aligned silk fibroin nanofibers with a diameter of 400 nm exhibited a better ability to direct MSC migration than the fibers with diameters of 800 nm and 1200 nm.^33^ The size of the electrospun fibers used for facilitating the cell migration should be optimized based on the intended application and the type of cells.

### D. The impact of fiber modulus on cell migration

The fiber modulus is another critical factor that affects cell migration. *In vivo*, tissue modulus ranges widely from 0.5 kPa (adipose tissue) to 20 MPa (bone) and changes both during development and in different diseases.^39^ Numerous studies have demonstrated the role of substrate modulus in regulating cell motility.^4,40–44^ When nanofibers are used as substrates, the migratory behavior of cells can also be regulated by the nanofiber modulus. In one study, fibrous mats with different surface moduli were fabricated using the co-axial electrospinning method.^45^ Different polymers [i.e., gelatin, poly(ethersulfone) (PES), poly(dimethylsiloxane) (PDMS), and PCL] were employed as the core to modulate the fiber moduli, while PCL was used as the sheath to conserve surface chemistry. Figure 5(c) shows the migration speed of single glioblastoma cell on different fibrous mats. Cell migration was fastest on the nanofibers of intermediate modulus (i.e., ca. 11 *μ*m/h for PCL nanofibers with a modulus of ca. 8 MPa); slower migration speeds were observed on the nanofibers with low modulus (i.e., ca. 3.5 *μ*m/h for gelatin-PCL with a modulus of ca. 2 MPa) and high modulus (i.e., ca. 5.8 and 6.3 *μ*m/h for PES-PCL and PDMS-PCL, respectively, with moduli of both ca. 30 MPa). The sensitivity of cells to the fiber modulus can be attributed to a cellular sensing process often referred to as the “catch-bond formation” mechanism,^46^ by which larger traction forces generated by cells can be evoked to promote their migration.

### E. The role of surface chemistry in controlling cell migration on fibers

The surface chemistry of the fibers also affects the migratory behavior of cells because cell migration is a balance between cell adhesion and cell movement on the fibers. While weak adhesion is not sufficient to stabilize the cell front, leading to impaired motility, overly strong adhesion also results in impaired motility due to the inability of the cells to break rear adhesion.^47^ Thus, optimal focal adhesion turnover (i.e., assembly and disassembly of adhesion) is required for good cell motility. Bioactive agents such as proteins and growth factors can be coated on the surface of the fibers through physical adsorption, ionic bonding, or covalent bonding to tune the interactions between the cells and the underlying fibers. For example, when laminin was grafted onto the surface of electrospun PLGA nanofibers, the migration of Schwann cells was greatly accelerated.^48^

**FIG. 6.**
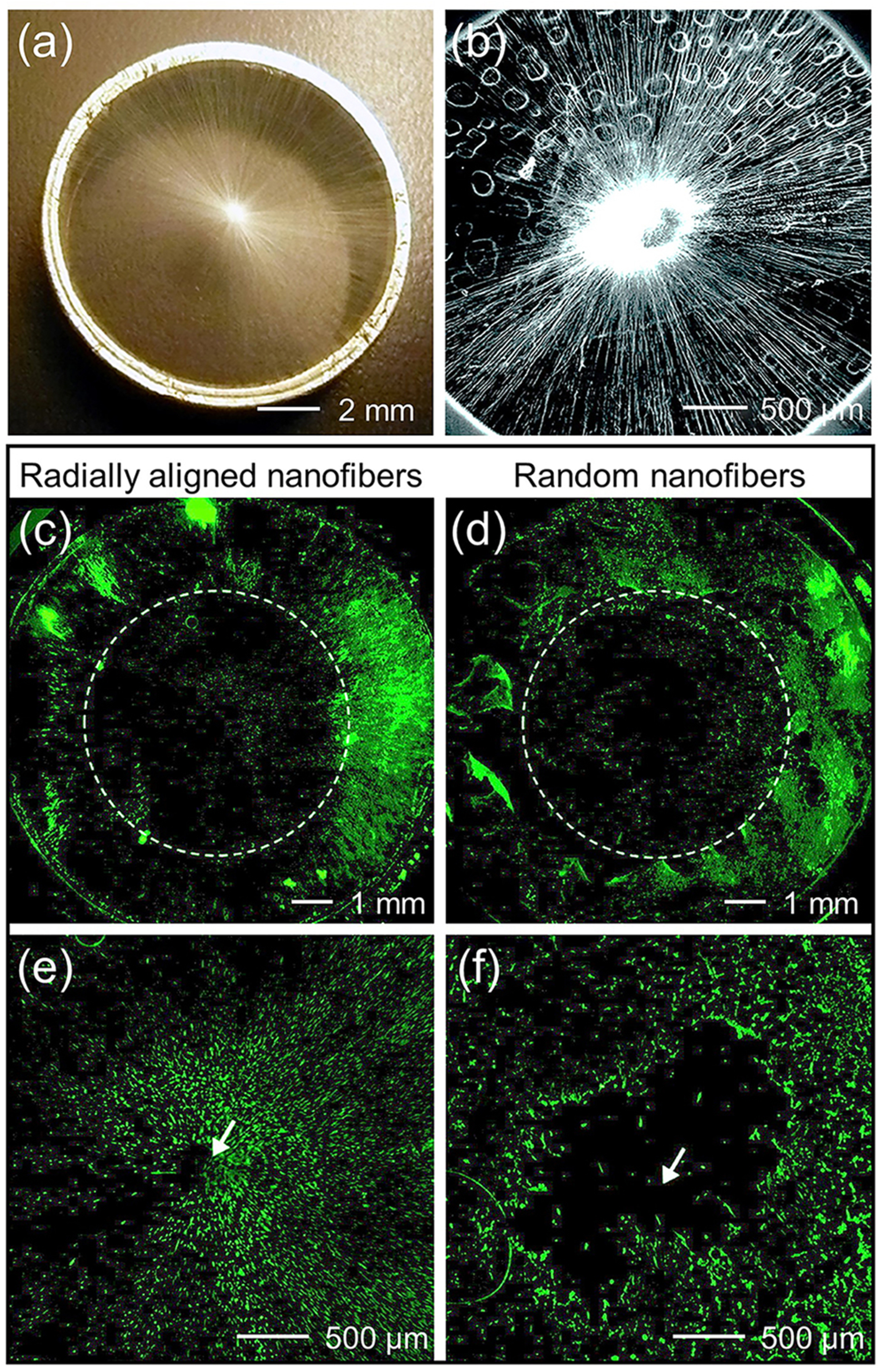
Migratory behavior of cells on radially aligned nanofibers. (a) Photograph and (b) SEM image showing the radial alignment of PCL nanofibers in a scaffold that was directly deposited on the ring collector. Fluorescence micrographs showing the migration of dura fibroblasts on scaffolds made of [(c) and (e)] radially aligned and [(d) and (f)] random PCL nanofibers from the periphery of the scaffold toward the center after four days of incubation. Enlarged views of the center portions in (c) and (d) are shown in (e) and (f), respectively. The arrow marks the center of the scaffold.^49^ Reprinted with permission from Xie *et al*., ACS Nano **4**, 5027 (2010). Copyright 2010 American Chemistry Society.

In addition to migration along the uniaxial direction, in some cases, it is desirable to induce the radial migration of cells from the periphery toward the center or vice versa. For instance, to facilitate the regeneration of dura mater after a neurosurgical procedure, the synthetic dural substitute must favor the adhesion of dural fibroblasts and simultaneously promote their migration from the periphery of the substitute toward the center. Radially aligned fibers have shown promise for this purpose.^49^ A collector consisting of a central point electrode and a peripheral ring electrode was developed to collect nanofibers oriented in a radial fashion. Figures 6(a) and 6(b) show a photograph and SEM image of PCL nanofibers, respectively, in a scaffold that was directly deposited on the ring collector, indicating the radial alignment of the nanofibers. In an *ex vivo* model, dura tissues were separately cultured on scaffolds composed of radially aligned nanofibers and random nanofibers to allow the migration of dural fibroblasts from the surrounding tissues to the scaffolds. After incubation for four days, the entire surface of the scaffold composed of radially aligned nanofibers was covered by the cells [Figs. 6(c) and 6(e)]; by contrast, a void was observed on the scaffold made of random nanofibers [Figs. 6(d) and 6(f)]. After the surfaces of the radially aligned nanofibers were coated with fibronectin, the adhesion and migration speed of the cells were further enhanced. As such, radially aligned nanofibers were able to interface with natural dura and promote the migration of host cells along the nanofibers. In another study, the migration of human MSCs from the periphery toward the center was accelerated on radially aligned nanofibers compared to that on random nanofibers.^50^ Modifying the nanofiber’s surface with polydopamine further enhanced the migration speed of the cells by presenting a favorable surface for cell adhesion.

Compared to a uniform coating of bioactive agents on the surface of nanofibers, a gradient cue on the nanofibers is more effective for accelerating cell migration along the direction of concentration increase. This occurs via the haptotaxis of cells in response to migratory cues, including graded adhered agents on the surface or anchored factors within the substrate. The first response of cells to a gradient is polarization, by which chemosensory signaling receptors are redistributed. If cellular polarization persists in one direction, cell migration will occur in succession.^51^ The variations in adhesion strength between the cells and graded cue on the fibers also attribute to cell migration. When the cells attach to the fibers more tightly at one end, the imbalance in adhesive force will lead to the movement of cells toward the direction of increasing adhesion.^52^

Various strategies have been used to integrate bioactive agents with concentration gradient on nanofibers. The simplest way to generate a gradient is to vary the contact time of the bioactive agent on the nanofiber’s surface along the direction of fiber alignment.^53^ In one study, a gradient of vascular endothelial growth factor (VEGF) over 1.125 mm was constructed on the surface of uniaxially aligned hyaluronic acid nanofibers by overlaying a microfluidic gradient generator on the nanofibers.^28^ It was found that the motility of human umbilical vein endothelial cells was maximized in the presence of the VEGF gradient along the direction of fiber alignment. Interestingly, when nanofibers were aligned perpendicular to the VEGF gradient, no effect of the VEGF gradient was observed, indicating that topographical cues were more influential than chemical cues in this case. A major drawback of this approach is that it requires a considerable volume of solution containing the expensive bioactive agent to generate a large-area gradient along the nanofibers. Additionally, the conditions for generating the gradient need be scrutinized for different types of bioactive agents and nanofiber compositions.

**FIG. 7.**
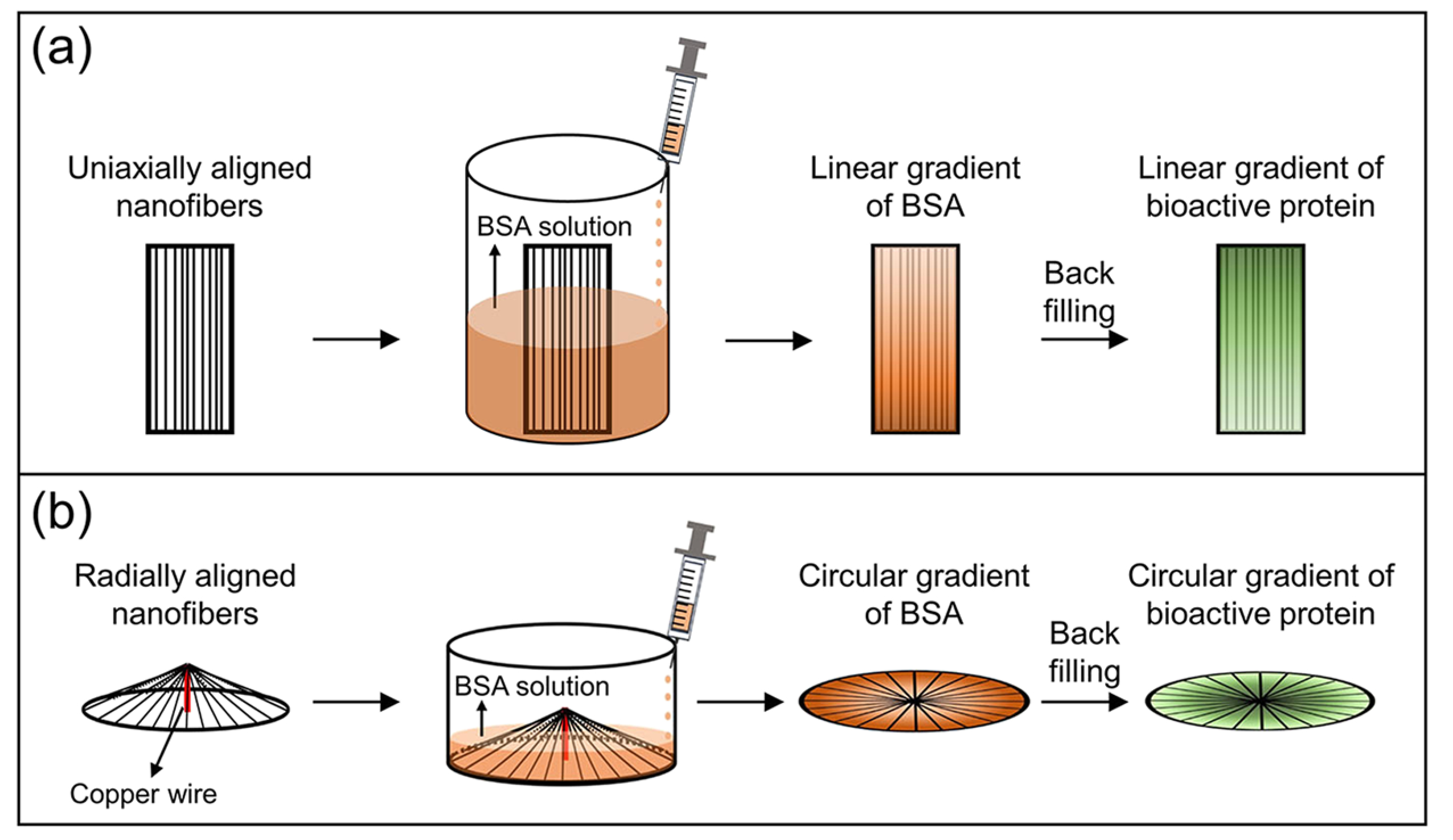
Generation of graded cues on the surfaces of aligned electrospun nanofibers. (a) Schematic showing the generation of a linear gradient of bioactive protein along uniaxially aligned nanofibers using BSA as the mask protein. (b) Schematic showing the generation of a circular gradient of bioactive protein along radially aligned nanofibers.^55^ (b) Reprinted with permission from Wu *et al.*, ACS Appl. Mater. Interfaces **10**, 8536 (2018). Copyright 2018 American Chemistry Society.

To solve these problems, a simple method was developed for creating gradients through a graded mask of bovine serum albumin (BSA).^54^ As shown in Fig. 7(a), this process involves two major steps. First, to serve as a mask, BSA is deposited on the nanofiber’s surface with a gradient by varying the immersion time of the nanofibrous mat in the BSA solution along one axis. Second, after pipetting a solution of bioactive protein onto the surface of the resulting nanofibrous mat, the bioactive protein fills the bare regions left behind on the surface to generate a gradient that runs countercurrent to the BSA gradient. This method was also applied to generate a circular gradient of bioactive protein on radially aligned nanofibers along the direction of fiber alignment, as shown in Fig. 7(b).^55^ By constantly increasing the volume of the BSA solution introduced into a container with a center-raised fibrous scaffold placed in the upright or upside-down configuration, a divergent or convergent BSA gradient was generated along the radially aligned nanofibers, respectively. A countercurrent, circular gradient of bioactive protein could then be produced. Compared to the scaffold composed of nanofibers uniformly coated with laminin, the circular gradient of laminin, which increased in concentration from the periphery to the center along the radially aligned PCL nanofibers, significantly accelerated the migration of fibroblasts from the periphery toward the center. Similar results were observed for the migration of keratinocytes on scaffold composed of radially aligned PCL nanofibers with a circular gradient of the epidermal growth factor on the surface.

Bioactive agents can also be immobilized on the surface of nanofibers based on high-affinity interactions in the presence of specifically recognized domains. For example, collagen-binding domain (CBD)-fused proteins can be selectively immobilized on the collagen domains of PCL-collagen blend nanofibers.^56^ By applying the ring collector to fabricate radially aligned nanofibers for a certain collection time, the nanofiber density can be controlled to gradually increase from the periphery to the center. After immobilizing CBD-fused stromal cell-derived factor-1α (SDF1α) in the collagen domains of the PCL-collagen blend nanofibers, a continuous gradient of SDF1α was formed along the radially aligned nanofibers, leading to the accelerated migration of neural stem cells from the periphery toward the center.

In addition to forming single layers of bioactive agents on the surfaces of nanofibers, bioactive agents can also be constructed into particles or immobilized on particles and then deposited onto the surface of nanofibers with a gradient. A linear gradient of protein-encapsulated PLGA microparticles was generated across a glass substrate by spatially varying the deposition time (during which the substrate was exposed to the electrosprayed microparticles).^57^ Using a scaffold made of uniaxially aligned nanofibers as the substrate, this strategy can be further applied to manipulate cell migration.

### F. The stimulus of cell migration by a soluble bioactive agent released from the fibers

The controlled, localized release of soluble bioactive agents from nanofibers is another important tool to encourage cell migration by taking advantage of the chemotaxis of cells. Chemotaxis is a phenomenon that enables cells to sense the concentrations of certain chemical species in their microenvironment by comparing the asymmetric activation of the receptors at the different ends of the cell and move toward chemically favorable regions.^7,58,59^ For example, leukocytes are drawn to the site of injury by chemotactic influences. Bioactive agents can be functionalized in scaffolds composed of aligned fibers through various means, such as directly blended in the nanofibers,^60^ contained within the core of core-sheath nanofibers,^61^ and included within microspheres that are co-electrospun to the surface or within the fibers.^62^ Depending on the encapsulation method of the bioactive agents and biodegradation rate of the fibers, release profiles ranging from an immediate burst release to sustained release over the course of several weeks can be realized. For example, the release of recombinant human VEGF from polyethylene oxide-PCL core-sheath fibers could significantly improve the migration of endothelial cells compared to plain nanofibers.^63^ By incorporating Cu_2_S particles into nanofibrous mats made of a blend of poly-*DL*-lactic acid and PCL, Cu ions could be released from the nanofibers, significantly accelerating the migration of endothelial cells.^64^ These studies collectively indicate that, by tailoring the properties of the electrospun fibers, the migratory behavior of different types of cells can be manipulated, as summarized in Table I.

## IV. MANIPULATING CELL MIGRATION FOR TISSUE REPAIR

The manipulation of cell migratory behavior using electrospun nanofibers has been applied to tissue repair. Unlike cell migration on a surface, cells must overcome the biophysical resistance of their microenvironment, including the high matrix stiffness and limited pore size in order to move *in vivo*.^65–67^ To promote tissue repair, it is necessary to facilitate the directional migration and proliferation of reparative cells at the wound site. To this end, in one study, a tri-component nanofibrous scaffold was developed, which was able to release multiple factors in a temporal sequence to direct cell migration for connective tissue repair in a meniscus damage model.^68^ Water-soluble poly(ethylene oxide) nanofibers were used as a sacrificial fraction to first deliver a burst of matrix-degrading enzyme (i.e., collagenase) to the wound interface in an effort to reduce local ECM density and stiffness, thus allowing cell migration and increasing interfacial cellularity.^69^ The slower-degrading hyaluronic acid nanofibers then delivered a chemoattractant (i.e., platelet-derived growth factor-AB), providing an exogenous chemoattractive gradient to recruit cells to the wound site and accelerate repair. The remaining population of stable PCL nanofibers acted as a physical template to provide mechanical integrity and instruction for organized ECM synthesis upon cell arrival. Even though random nanofibers were utilized in this study, it is promising to extend this concept to aligned nanofibers to foster tissue formation that recapitulates the structural and mechanical anisotropies of the native organized connective tissues.^70^

## V. MANIPULATING CELL MIGRATION FOR CANCER TREATMENT

**FIG. 8.**
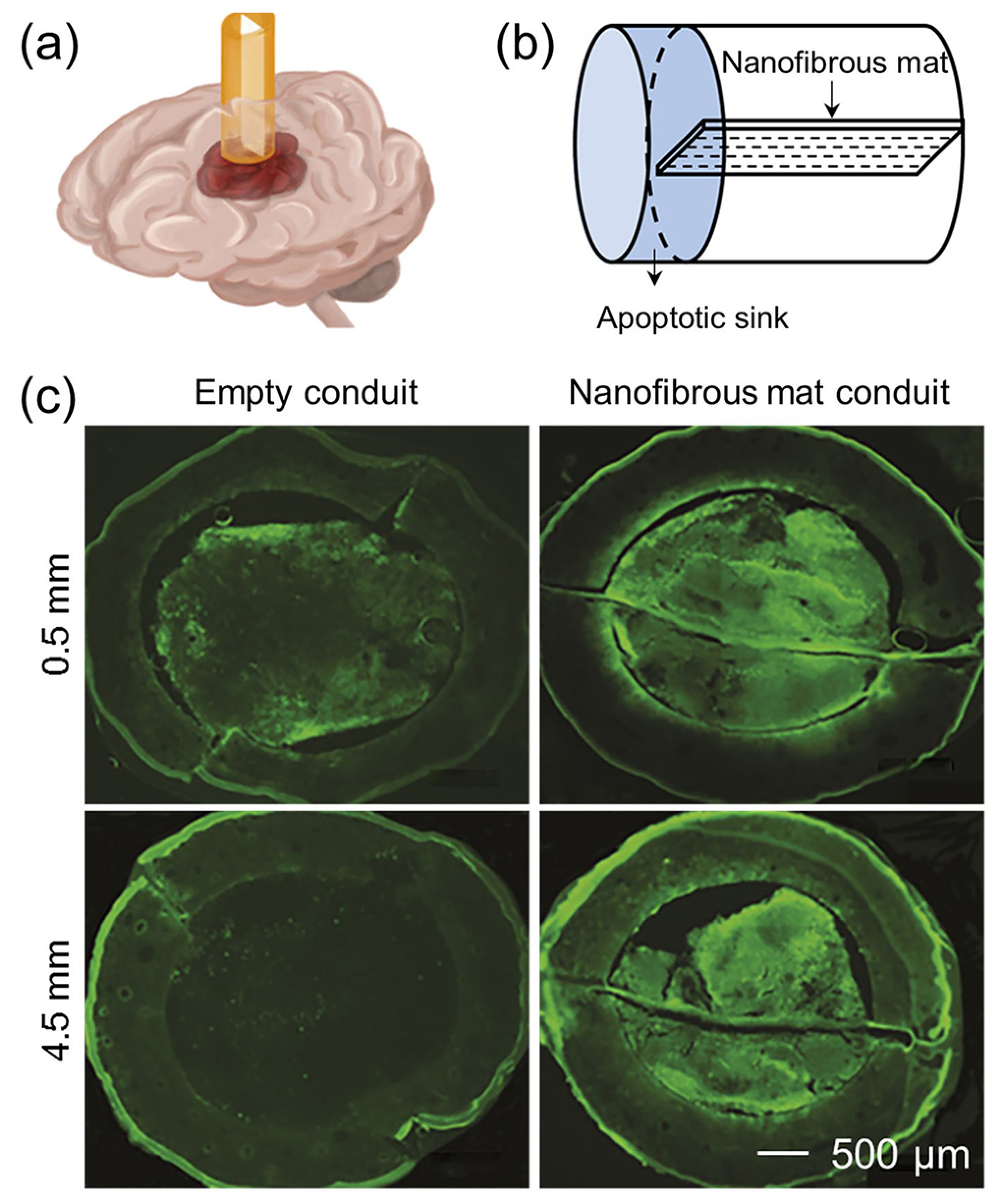
[(a) and (b)] Schematic showing that a conduit containing a uniaxially aligned nanofibrous mat was inserted into a rat brain for guiding the migration of glioblastoma cells from the primary tumor site into the apoptotic sink. (c) Fluorescence micrographs showing the coronal sections at 0.5 and 4.5 mm along the empty conduit and the conduit containing the nanofibrous mat from the tumor core. More tumor cells migrated into the conduit containing the nanofibrous mat than that into the empty conduit.^73^ Reprinted with permission from Jain *et al.*, Nat. Mater. **13**, 308 (2014). Copyright 2014 Springer Nature.

The manipulation of cell migratory behavior using electrospun nanofibers has also been applied to cancer treatment. The electrospun nanofibers can be used to construct models that accurately mimic the microenvironment of invading cancer cells to better understand the migration of cancer cells and test potential anti-migration therapies of cancer cells. Cancer is a disease of uncontrolled cell growth along with uncontrolled cell migration.^71^ A broad spectrum of migration mechanisms can be involved for both the individual and collective migration of cancer cells.^6,72^ Electrospun nanofibers have been applied as substrates to examine the migration behavior of cancer cells (e.g., glioblastoma multiform cells and breast cancer cells).^38,45^ Furthermore, electrospun nanofibers can serve as “tumor guides” to move primary intracorticals tumor to extracortical locations, making it possible to “manage” the growth of tumors by giving them a preferred and pre-specified escape route. To this end, a tumor guidance conduit was constructed from two components: a PCL-polyurethane conduit containing a uniaxially aligned PCL nanofibrous mat with a thickness of 10 *μ*m, and a cyclopamine-conjugated collagen hydrogel that served as an apoptotic “tumor sink.”^73^ As shown in Figs. 8(a) and 8(b), one end of the conduit was implanted near the tumor boundary, and the other end containing the cyclopamine collagen gel was located above the skull surface. Figure 8(c) shows the *in vivo* migration of tumor cells in the empty conduit and the conduit containing the nanofibrous mat. Compared with the empty conduit, more tumor cells migrated into the conduit containing the aligned nanofibrous mat, and tumor cells were distributed along the entire length of the conduit. After guiding the invasive tumor cells away from the primary tumor site to the extracortical collagen hydrogel, the tumor cells underwent apoptosis, and the tumor volume in the brain was also significantly reduced.

## VI. PERSPECTIVE AND PROSPECTS

In conclusion, electrospun fibers are capable of manipulating cell migration, and this capability can be tailored and optimized for applications in tissue repair and cancer treatment. For these applications, it is necessary to take a full consideration on the desired properties of the fiber substrate, the required bioactive agents, and the specific function and structure of the target tissue to optimize the migration and recruitment of cells.

Along with the developments toward the electrospinning technique, there are also many forward-looking aspects in applying the aligned fibers to manipulate cell migration. While the alignment, size, composition, mechanical strength, and surface chemistry of the fibers have all been tailored to manipulate cell migration, the following potential developments are envisioned in the near future.

- Endowing electrospun fibers with electrical signals. Electrotaxis plays an important role in regulating cell migration. Motile cells exposed to an external DC electric field will reorient and migrate along the direction of the electric potential.^74^ For instance, as a prime directional cue, electric fields at strengths equal to those detected endogenously at wounds have been reported to direct cell migration during wound healing.^75–77^ Clinically, for wound healing management, it is highly desirable to develop reliable technologies that exploit electric signaling.^76^ Therefore, combing electrospun nanofibers with an electrical signal is a promising method to promote cell migration and thus tissue repair.
- Integrating different types of cues into one scaffold to optimize cell migration and thus tissue repair. It is important to note that cells engaged in directed migration *in vivo* likely encounter multiple types of cues that they must simultaneously evaluate and prioritize to achieve an appropriate physiological response. Chemotactic, haptotactic, durotactic, and galvanotactic cues all play important roles, and these cues also affect each other. For example, chemotaxis can be modulated by substrate stiffness; higher cell chemotaxis is observed on softer substrates.^78^ To construct a niche for facilitating cell migration and tissue repair, an ideal scaffold should possess characteristic features of natural ECMs to allow different types of cues to act in concert. The architecture, composition, size, and mechanical strength of the electrospun fibers should all be carefully adjusted to achieve multi-functionality. Moreover, rather than two-dimensional mats, three-dimensional scaffolds with aligned topographical cues are desirable for tissue repair. A gas-foaming technique followed by freeze-drying has been developed to generate three-dimensional electrospun fibrous scaffolds with highly ordered structures.^79^ This technique can be further combined with the construction of graded surface to support cell migration in three-dimension. Furthermore, apart from sensitization to one type of growth factor, cell chemotaxis can be promoted by the synergy of multiple chemokines. The controlled release of multiple bioactive agents in a defined sequence from the scaffold is highly promising for promoting cell migration. In addition, during tissue repair, more than one type of cell migrates toward the defect site. Current studies are mostly restricted to one type of cell line. Thus, it is necessary to establish a system that incorporates multiple types of cells to involve the role of cell-cell interactions during cell migration on fibers. A better understanding of the mechanism of cell migration is also required to instruct the design of fibrous scaffolds.
- Simultaneously manipulating the migration and differentiation of stem cells using electrospun fibers. Manipulating the migration of stem cells is of great importance for accelerating tissue repair and controlling cancer metastasis. Due to the multi-lineage differentiation potential of stem cells, the properties of the fibers affect not only the migration but also the phenotype of the cells.^80^ Any uncontrolled differentiation of stem cells will be a disaster. Therefore, when using electrospun fibers for manipulating the migration of stem cells, it is crucial to simultaneously evaluate and control the differentiation of stem cells.

Although electrospun fibers have shown great potential for manipulating cell migration, the electrospinning technique still faces a set of challenges. For example, it is still difficult to precisely control the structural parameters of electrospun fibers. The diameters of the fibers are typically distributed over a certain range. To ensure both homogeneity and reproducibility for the fibers, it is necessary to precisely control the electrospinning parameters, as well as the properties of the polymer solution during an electrospinning process. Currently, the fabrication of aligned fibers is mainly restricted to the laboratory setting in a relatively small volume due to the use of a single spinneret only. Although industrial lines based on multiple needles or needleless electrospinning have enabled the production of electrospun fibers on a large scale, it still remains a challenge to continuously collect the fibers with well-controlled alignment. In addition, when the fibers are electrospun from polymer solutions, it is required to completely remove any residual solvent in the fibers to avoid the potential toxicity to cells and human body. These challenges also represent the future directions and opportunities to push forward the development of electrospinning technique. The ultimate goal is to move the electrospun fibers toward applications from laboratory to industry and from bench to bedside.

## Figures and Tables

**TABLE I.** Manipulation of cell migration by tailoring the different parameters associated with the electrospun fibers.

Parameter	Material	Cell type	Method	Outcome	References
Alignment	Thermoplastic polyurethane fibers	3T3 fibroblasts	Wound assay	The migration speed of cells cultured on uniaxially aligned nanofibers was approximately two times that of cells cultured on random nanofiber	30
Diameter	PLA fibers	Schwann cells	Cell tracking	Schwann cells migrated the farthest on the large fibers (1325 ± 383 nm) and the shortest on the small nanofibers (293 ± 65 nm)	37
Modulus	PCL nanofibers; core-sheath gelatin-PCL, PES-PCL, and PDMS-PCL nanofibers	Glioblastoma cell	Cell tracking	On the nanofibers with intermediate modulus (i.e., PCL nanofibers with a modulus of ca. 8 MPa), the cells migrated the fastest	45
Surface chemistry	PCL-collagen blend nanofibers	Neural stem cells	Wound assay	A continuous gradient of stromal cell-derived factor-la along the radially aligned nanofibers accelerated the migration of neural stem cells from the periphery toward the center	56
Soluble bioactive agents	Polyethylene oxide-PCL core-sheath nanofibers	Endothelial cells	Trans-well migration assay	The release of recombinant human VEGF from the nanofibers promoted the migration of endothelial cells	63
